# Impacts of access to legume- or grass-based pasture on behaviour, physiological responses and bacterial load of laying hens

**DOI:** 10.1016/j.heliyon.2024.e34780

**Published:** 2024-07-18

**Authors:** O.E. Oke, O.M. Onagbesan

**Affiliations:** a Animal Physiology Department, Federal University of Agriculture Abeokuta, Nigeria; b Centre of Excellence in Avian Sciences, University of Lome, Togo

**Keywords:** Environment, Hen, Pastures, Welfare, Behaviour

## Abstract

Despite the plethora of studies on the impacts of access to runs on chickens, there is a paucity of information on the welfare and behavioural repertoire of hens raised in the deep litter houses with or without access to legume- or grass-based pasture. Therefore, this study aimed to evaluate the impact of access to grass or legume pastures by laying hens on behaviour, physiological responses and bacterial load. The study was conducted to evaluate the influence of exposure of egg-type chickens to runs on grass or legume pastures on their welfare and behaviours. The study involved the use of 240 ISA brown pullets from 12 weeks of age and and lasted for 48 weeks. The treatments were deep litter housing with grass-based pasture run (PG), deep litter housing with legume-based pasture run (PL) and deep litter housing without runs (LD) having 80 pullets with four replicates of twenty birds each. Behavioural observations of the hens in each pen were made at 52 weeks of age and tonic immobility was assessed by making the birds lie on their back with their head resting in a U-shaped wooden cradle. The measurements of the respiratory rate and rectal temperature of the hens were assessed at 1:00 p.m. at different laying phases. The gastrointestinal and egg bacterial counts were conducted at 60 weeks of age. Results revealed that the proportion of time spent eating was highest (p < 0.05) in the deep litter housing system, while the legume and grass pasture were similar. The hens spent most of their time standing and eating in the three treatments. However, the time spent standing in PL and PG was similar but significantly higher (p < 0.05) than in LD. Results on tonic immobility duration showed that the time spent by the hens in LD during the reaction was significantly longer than those of the PL and PG in the first, second and third phases of the experiments. However, the time spent by the hens in PL and PG was similar. The rectal temperatures of PL and PG birds were comparable and higher than those of LD during the second phase. On the other hand, there was no difference in the respiratory rate. Plasma triiodothyronine (T_3_) of the hens did not follow a consistent pattern. The bacterial count in the large intestine in LD and PL was similar but significantly (P < 0.05) higher than that of the PG. It was concluded that access to pasture influenced the behaviours of hens and that tonic immobility duration was shorter in the hens on the pasture, suggesting that access to pasture favoured hens’ welfare.

## Highlights

• Access to grass or legume pastures significantly affected laying hen behaviours.
• Hens with access to pastures show reduced tonic immobility duration, indicating improved welfare.
• Rectal temperature was higher in hens with pasture access during specific phases.
• Bacterial load on the eggs was higher in the hens with access to pasture.
• Legume or grass pastures access enhanced hen welfare.

## 1 Introduction

An increasing number of consumers are paying more attention to how food is produced, as evidenced by the global trend toward food items with organic or animal welfare certifications. The condition of chickens kept for eggs in typical commercial cages has come under close examination. For some consumers, a greater level of poultry welfare through alternative technologies is crucial [[1], [2], [3], [4]]. Although the conventional housing of layers in traditional cages has been considered the most effective way to house laying hens; , it is now generally believed to have a detrimental impact on the hens' welfare. Additionally, the concerns about the use of antibiotics, such as drug resistance and residues in animal tissues, have increasingly come to the public's attention in recent decades [[5], [6], [7], [8], [9], [10]].

Concerns for the well-being of hens kept in traditional cages have led to modifications in housing across Europe [11,12]. There is a phase-out of cage-based systems, and farmers must adhere to strict welfare requirements if they must be used [3]. The use of conventional cages has been prohibited since January 1st, 2012, in compliance with the directive of the European Union enacted in 1999 [3,13]. According to this order, all cages now in use must have 750 cm^2^/bird area and be enhanced with features that allow birds to exhibit their behaviours. The search for better housing systems for chickens has also led to the adoption of free range to allow the birds to exhibit their natural behaviours.

The natural behavioural repertoires of the present-day hens consist of ancestral behaviour propensities displayed when they are given access to diverse resources [14,15]. Studies have shown that the degree of the expression of behavioural patterns of hens depends on their housing, epigenetic effects, embryonic development, and rearing environment [[16], [17], [18]]. Hens are severely limited in their behaviour, with poor musculoskeletal systems, and have positive affective conditions impaired in conventional cages [19].

The main concerns of consumers about farm animal care on housing and stocking conditions have been documented [11,20,21]. Cages prevent hens from engaging in the majority of their natural behaviours, such as freely walking, stretching, flying, jumping, running, exercising, foraging, scratching, dust-bathing, perching and nesting. This raises welfare concerns because it prevents hens from expressing certain behaviours that they are motivated to engage in, which can cause emotional stress or the development of abnormal behaviours, like pecking of feathers. Due to lack of activity, using cages also increases the risk of severe disuse osteoporosis. Hens can wander around their habitat freely and engage in the majority of the behaviours that are inhibited by cage confinement in alternative, cage-free setups.

It is generally believed that robust animal welfare encompasses the presence of positive experiences apart from the absence of negative experiences and health problems [22]. Numerous indicators are necessary to understand animal welfare and its assessment [23,24]. Several indicators can be used to assess hens' welfare, including plumage condition, heterophil/lymphocyte ratio, behaviour repertoire, bone strength, fearfulness (tonic immobility), thyroid hormones, rectal temperature and respiratory rate, among others [[25], [26], [27], [28]].

The gastro-intestinal tract of chickens houses a variety of bacterial species (gut microbiota) [29], which is crucial for physiological activities like production, overall health, nutrient absorption and digestion [30]. The gut microbial composition can be influenced by housing environments. Several studies have emphasized the potential effects that variations in the housing environment may have on the diversity and populations of the gut microbiota [[31], [32], [33], [34]] and microbial growth on eggshell [35].

Several attempts have been made to develop suitable alternatives to the conventional production systems. For instance, the use of enriched cages that permit hens to express natural behaviours, including nesting, roosting, and scratching was prompted by worries about the wellbeing of laying hens bred in battery cages [15,36,37]. Studies have been intensified on different ways of improving the welfare of chickens [[38], [39], [40], [41], [42], [43]]. However, there is a scarcity of information on the bacterial load, welfare and behaviour of laying hens reared on deep litter with different pasture types. Our earlier study showed that the reproductive and production performance of hens on legume-based pastures was better than that of grass-based pastures [38]. However, it is not clear if access to different pasture types could translate to better welfare for the birds. We hypothesized that access to free runs on legume or grass-based pasture would improve the bacterial load, behaviour and welfare of hens. The present study, therefore, sought to investigate the effect of access to grasses or legume pastures on the welfare and behaviour of laying hens.

## 2 Materials and methods

### 2.1 Ethical approval

The trial was approved by the Institutional Animal Ethics Committee guidelines of the Federal University of Agriculture, Abeokuta, Nigeria and that of the Federal Republic of Nigeria (FUNAAB/COLANIM/ACUC/2022/0007). The hens were given adequate management and care without unnecessary discomfort during the trial.

### 2.2 Paddock establishment

Before the paddock was established, the experimental plots were ploughed and harrowed. *Cynodon dactylon* was established using the sprigs. Seven m^3^/ha of the sprigs were planted in a properly prepared seedbed. *Stylosanthes hamata* seeds, which were planted at a rate of 3 kg of seed/ha, were used to establish the legume pasture. During the dry season, irrigation was used to maintain the pasture.

### 2.3 Experimental chickens and management

A total of two hundred and forty Isa Brown pullets were obtained from a reputable commercial farm (Animal Care, Ogere, Nigeria) and used for this study. The study was carried out at the Research Farms of the Federal University of Agriculture, Abeokuta, Nigeria. The birds were housed in three experimental groups at the age of 12 weeks: deep litter without runs (LD), deep litter with access to a pasture of grass (PG), and deep litter with access to a pasture of legumes (PL). Each replicate's pasture measured 80 m^2^ in size. There were four replicates of twenty birds for each treatment. Conventional feeders and drinkers were used in all the treatments. The nest boxes used were constructed with wood and had the dimension of 0.90 × 0.40 × 0.60 m (L × W × H). The birds in each of the treatment groups were provided with the same space of 15 cm perch per bird at a height of 45 cm.

Wood shavings at 8–10 cm depth were used as beddings. Production management procedures for poultry were implemented on a regular basis. The litter was changed as needed, feeders and drinkers were cleaned, and the layers' medication and vaccination schedules were meticulously followed. Water and concentrate feed were available at all times. The conventional layer diet was used [40]. The experiment lasted for one year. The chickens were exposed to 16 h of light per day. The average relative humidity and temperature during the study were 82 % and 34ᵒC, respectively.

## 3 Data collection

### 3.1 Behavioural observations

Birds in each replicate had their behaviours observed at 52 weeks of age for seven days. The description of each behaviour [44] is shown in Table 1. These behavioural recordings were carried out by observers who were positioned in front of each replicate, with the first 5 min used to adapt the hens to their presence. Following the adaptation period, each observer continuously performed 25 min of focal sampling on two randomly chosen birds per pen. The hens were chosen at random and marked with a non-toxic marker at least one week prior to data collection.

**Table 1:** Description of behaviours of laying hens.

Behaviour	Description
Eating	Picking the feed from the feed trough and pasture
Drinking	Ingestion of water from the drinker
Standing	Standing still posture or alert in one place
Walking	Taking at least one step in any direction
Sitting	Sitting with head retracted and eyes open or closed
Pecking	Gentle pecks aimed at beak, at particles in the body of another bird; pecking at the perches, trough and cages; pecking at claw, tail, head, neck and feathers, of another bird;
Panting	Panting in any posture
Nesting	Behaviour exhibited in the nest
Perching	Behaviour expressed on the perch
Other behaviours	Severe aggression, lying, wing stretching and flapping, and preening

### 3.2 Tonic immobility

Tonic immobility was carried out during the three laying (early, peak and late) phases. Immediately a bird was gently captured, the inducement of tonic immobility was done by laying the bird on its back with their head resting in a wooden U-shaped cradle, as described by Ref. [45]. For 10s, the bird was gently restrained. Due to the fear-inducing effects of eye contact, the observer sat in full view of the chickens, about 1 m away, and fixed his or her eyes on the chicken. After the experimenter had removed his or her hands and the bird had been motionless for 10 s, a stopwatch was started to time how long it took the bird to right itself. The restraint process was repeated if the bird righted itself in less than 10 s because tonic immobility was deemed not to have been induced. The highest score of 900 s was awarded for righting time if the bird did not exhibit a righting reaction during the 15-min testing time.

### 3.3 Rectal temperature

Every week at 1 p.m. for each treatment, the rectal temperatures of two birds per replicate were recorded. A digital thermometer (TM-747DU, Tenmars Electronics Co., Ltd., Taipei, Taiwan, having ± 0.1 °C accuracy was gently inserted into the rectum. Each bird was gently and patiently restrained, and the thermometer beeped until the reading was complete.

### 3.4 Respiratory rate

With the use of a stopwatch, the movement of the vent or the abdomen region was counted, as described by Ref. [46], to determine the respiratory rate of two birds per replicate weekly.

### 3.5 Plasma triiodothyronine

Blood samples were collected from 2 hens per replicate weekly for the determination of the plasma triiodothyronine (T_3_). T_3_ was determined using a commercially available chicken ELISA kit (MyBioSource, Inc., San Diego, CA). This procedure used a microplate reader capable of readings at 450 nm wavelength. Moreover, a software package was used to facilitate data generation, analysis, reporting and quality control. The analyses were performed essentially as described in the manufacturer's manuals. Each sample was prepared in duplicate to enhance precision.

### 3.6 Bacterial count on eggs and gastrointestinal tract

A total of sixteen (16) birds per treatment (4 per replicate) were selected from each of the treatment groups at forty weeks. The eggs were collected using sterile hand gloves. After slaughtering the birds, digesta samples were collected from the crop, small intestine, caeca and large intestine for a microbial count. Nutrient agar powder was weighed according to manufacturer specifications (28 g/L). It was soaked in distilled water for 15 min and sterilized at 126 °C for 11 min. After cooling to room temperature, it was used for the inoculation of samples. The samples were serially diluted to different decimal dilutions under aseptic conditions, and 1.0 ml of the dilutions was aseptically inoculated on freshly prepared nutrient agar plates using the pour plates’ method. The samples were inoculated in duplicates. Incubation was conducted at 37 °C for 48 h. The number of colonies was counted after the incubation period. The bacteria colony on the plates was further subcultured on nutrient agar plates until the pure culture was obtained. With the aid of visual inspection, clean eggs of average size were sampled from each of the treatments and the procedure of [47] was adopted for the assessment.

### 3.7 Statistical analysis

The data collected were subjected to a one-way Analysis of Variance and Tukey's test was used to separate the means using [48] statistical program. The significance level was set at P < 0.05. The results are presented as Mean ± Standard Error of Mean.

## 4 Results

### 4.1 Behaviours

**Fig. 1:**
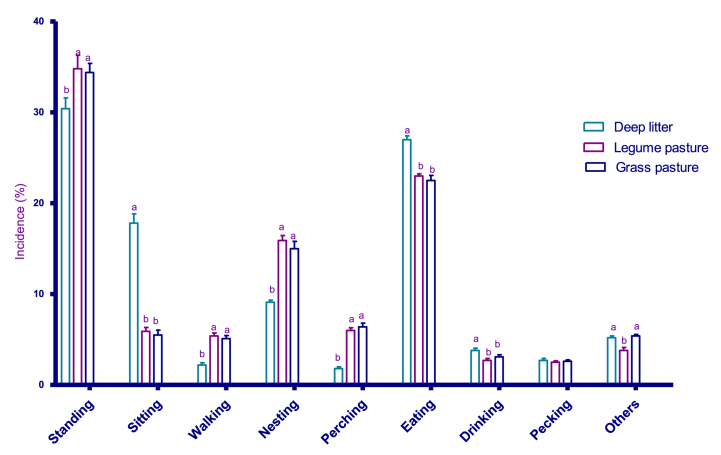
Effects exposure to pasture on behavioural incidence of hens. Means within one of the behaviours with different superscript letters differ significantly (P < 0.05); the error bars on the bar chart represent the standard errors of means.

The behavioural incidence of laying hens is presented in Fig. 1. The proportion of time spent eating was highest (p < 0.05) in the deep litter housing system, while the legume and grass pastures were similar. The hens spent most of their time eating and standing in all three housing systems. However, the time spent standing in PL and PG was similar but significantly higher (p < 0.05) than in LD. Compared to chickens kept in PL and PG, LD had a higher incidence of sitting but a lower incidence of walking. The time spent drinking in LD was higher (p < 0.05) than those of PL and PG, while PL and PG were similar. In contrast, PL and PG were similar in the proportion of time spent perching but significantly higher than LD. The incidence of pecking was similar in all the housing systems, as there was no significant difference. Moreover, the proportion of time spent performing other behaviours (preening, flapping wings, lying and severe aggression) in LD was not different from that of PG but significantly higher than in PL.

### 4.2 Tonic immobility

Table 2 reveals the tonic immobility induction and duration in layers. There was no significant effect on the number of times required to achieve tonic immobility. Numerically, the results obtained for LD were, however, lower than those of PL and PG. For tonic immobility duration, the time spent by the hens in LD during the reaction was significantly longer than that of the PL and PG in the first, second and third phases of the experiments. However, the time spent by the hens in PL and PG was similar.

**Table 2:** Effects of exposure to pasture on the mean tonic immobility induction and duration in layers at different laying (early; peak; late) phases.

Rearing system	Laying phases	TI induction	TI duration (s)
**Deep litter**	Early	1.125 ± 0.13	753.75 ± 31.45^a^
	Peak	1.00 ± 0.00	739.87 ± 9.49^a^
	Late	1.00 ± 0.00	649.73 ± 9.65^a^
	P value	0.2963	0.0001
**Legume pasture**	Early	1.50 ± 0.18	195.88 ± 4.10^b^
	Peak	1.50 ± 0.27	239.43 ± 9.64^b^
	Late	1.25 ± 0.16	180.25 ± 4.34^b^
	P value	0.1692	0.0001
**Grass pasture**	Early	1.38 ± 0.18	196.87 ± 4.43^b^
	Peak	1.38 ± 0.18	231.37 ± 5.28^b^
	Late	1.38 ± 0.18	188.88 ± 5.42^b^
	P value	0.1875	0.0001

Means in a row with different superscript letters differ significantly (P < 0.05); TI = Tonic immobility; Data are presented as Mean ± Standard Error of Mean.

### 4.3 Rectal temperature and respiratory rate

Table 3 shows the effects of rearing systems on the rectal temperature and respiratory rate of layers at different laying phases. The rearing systems did not significantly influence rectal temperature and respiratory rate in the first phase of egg production.

**Table 3:** Effects of exposure to pasture on the mean rectal temperature and respiratory rate of layers.

Rearing system	Rectal temperature (^0^C)	Respiratory rate (pulse per minute)
**(18–32 weeks old)**		
Deep Litter	41.53 ± 0.05	22.82 ± 0.95
Legume pasture	41.35 ± 0.10	24.95 ± 1.18
Grass pasture	41.56 ± 0.08	24.95 ± 0.51
P value	0.1326	0.1968
**(33–46 weeks old)**		
Deep Litter	41.74 ± 0.05^b^	24.94 ± 0.54
Legume pasture	41.98 ± 0.05^a^	25.94 ± 0.34
Grass pasture	42.01 ± 0.07^a^	26.04 ± 0.69
P value	0.0056	0.3213
**(47–60 weeks old)**		
Deep Litter	41.81 ± 0.04	24.25 ± 1.38
Legume pasture	42.79 ± 0.02	25.38 ± 0.84
Grass pasture	42.58 ± 0.07	26.75 ± 0.59
P value	0.0001	0.2280

^ab^ Means with different superscripts differ within column (P < 0.05); Data are presented as Mean ± Standard Error of Mean.

In the second phase, the rectal temperature in PL and PG was similar and significantly higher than that of LD. There was, however, no influence on the respiratory rate.

In the third phase, the difference in rearing systems did not significantly affect rectal temperature and respiratory rate.

### 4.4 Triiodothyronine

The effect of rearing systems on plasma triiodothyronine (T_3_**)** concentrations in layers is presented in Table 4. The difference in the rearing system had no influence on the T_3_ level at week 15. At week 20, the levels of T_3_ in LD and PL were similar but significantly higher than that of PG. At week 25, the level in PG and PL were comparable but significantly higher than that of LD. However, in week 30, LD was significantly higher than PL and PG, while PL and PG were similar. There was no significant difference at week 40. At week 50, the level of T3 in PG was similar to that of PL but significantly higher than that of LD. At week 60, the level in PL was significantly higher than those of the PL and LD, while PL and LD were similar.

**Table 4:** Effect of rearing systems on mean plasma triiodothyronine (T_3_) levels (ng/ml) of layers.

Age (weeks)	Rearing systems			
	Deep litter	Legume pasture	Grass pasture	P value
15	2.86 ± 0.14	2.14 ± 0.18	3.12 ± 0.59	0.2050
20	3.49 ± 0.28^a^	2.50 ± 0.51^ab^	1.87 ± 0.03^b^	0.0227
25	2.49 ± 0.05^b^	2.99 ± 0.08^a^	2.89 ± 0.10^a^	0.0034
30	3.65 ± 0.27^a^	3.06 ± 0.06^b^	2.84 ± 0.13^b^	0.0251
35	2.76 ± 0.18	2.75 ± 0.07	2.54 ± 0.33	0.7508
40	2.95 ± 0.07	2.85 ± 0.19	2.91 ± 0.15	0.8980
50	2.77 ± 0.09^b^	3.97 ± 0.97^ab^	5.92 ± 0.53^a^	0.0209
60	4.46 ± 0.77^b^	7.17 ± 0.71^a^	4.65 ± 0.57^b^	0.0380

^ab^ Means with different superscripts differ significantly along the row (P < 0.05); Data are presented as Mean ± Standard Error of Mean.

### 4.5 Bacterial count

Table 5 shows the effects of rearing systems on bacterial count (cfu/ml) in egg-type chickens. The rearing systems significantly influenced the bacterial count on the egg surface. The count was higher (P < 0.05) in PL than in PG. The lowest count was recorded in LD. Conversely, the bacterial count in the crop in LD was higher than those in PL and PG. The counts in PL and PG were similar. The count in the large intestine in LD and PL was similar but significantly (P < 0.05) higher than that of the PG. Moreover, the count in PL was higher than that of PG but similar to that of LD. PG and LD were, however, comparable. The rearing systems did not influence the caecal count.

**Table 5:** Effect of exposure to pasture on mean bacterial count (cfu/ml) on eggs and digestive system of the chickens.

Parameter	Rearing system			P value
	Deep litter	Legume pasture	Grass pasture	
Egg surface	43.31 ± 1.13^c^	60.29 ± 1.48^a^	56.56 ± 0.64^b^	0.0001
Crop	5.28 ± 0.80^a^	2.75 ± 0.35^b^	2.68 ± 0.29^b^	0.0029
Large intestine	5.73 ± 0.86^a^	5.15 ± 0.43^a^	3.20 ± 0.38^b^	0.0180
Small intestine	5.08 ± 0.33^ab^	5.83 ± 0.64^a^	3.85 ± 0.40^b^	0.0251
Caecum	2.88 ± 0.32	2.38 ± 0.13	2.53 ± 0.10	0.2440

^ab:^ Means with different superscripts differ within rows (P < 0.05); Data are presented as Mean ± Standard Error of Mean.

## 5 Discussion

The complexity of the environment can influence the level of fear in chickens [[49], [50], [51]]. Fear is an important component of stress [52,53]. According to earlier reports of Campbell et al. and Carli, Farabollini [54,55], the variables of tonic immobility (TI) reaction, which is expressed as an unlearned state of diminished reactivity to external stimulus and caused by light physical confinement, are the most reliable indicators of fearfulness in chickens. The hippocampus is the centre that responds to the psychological and emotional disturbance caused by stress and is involved in the development and termination of tonic immobility [56]. In the present trial, the difference in the rearing systems slightly influenced the chickens' susceptibility to tonic immobility reaction (the ease of being induced) and their duration of TI, respectively. The duration of tonic immobility was considerably prolonged in the hens in the deep litter than in the pasture. This is consistent with the observation of Skomorucha et al. and Bari et al. [57,58], who indicated that chickens kept outdoors had better welfare than those raised indoors. The protracted TI duration indicated a higher level of fearfulness in this group and corroborates the earlier result on the H:L ratio as a measure of stress in the birds [42]. The difference in the fearfulness of the birds in the different treatments could be explained by the variation in the environmental set-up [59]. The greater fearfulness in the birds in the deep litter system could be attributed to a lack of environmental complexity, which provides extra stimulation that may affect birds’ expectations about their environment and enhance their adaptation. This agrees with the findings of Al-Aqil et al. and Campbell et al. [60,61], who reported that chickens kept in a closed chamber were more fearful than those in an open-sided housing system. The reports of Sossidou et al. and Vas et al. [62,63], indicate that the welfare of chickens could be enhanced if given access to runs. Additionally [64], indicated that the level of fearfulness was reduced by environmental complexity. In contrast to the findings obtained in this study, Campo et al. [65], reported no significant difference in the tonic immobility of the chickens reared on the free range and deep litter housing system. The discrepancy may be due to differences in the environmental conditions. The findings of Escobar et al. [66], also indicated that bedding materials did not influence fearfulness in broiler chickens. The similarity in the TI recorded for the hens in the legume and grass pasture can be attributed to their access to runs.

According to Tahamtani et al. [67], adding environmental complexity to a harsh conventional housing system encourages birds to engage in their natural behaviours, while enhancing their productivity, livability, and feather health and lowering their aggressive tendencies. Also, Guo et al. and Cheon et al. [68,69], reported that the rearing system can strongly influence the behaviour of hens. In this trial, the hens kept in deep litter without access to the pasture showed more sitting and less walking behaviour compared to grass and legume pasture (Fig. 1). This suggests that the hens in the free range are more active than those in confinement in the deep litter. Free-range birds have been reported to be more active [70]. In addition, Leyendecker et al. [71], found that free-range birds exhibit higher physical activity and opportunity for the expression of natural behaviour than the birds housed in traditional systems. Additionally, the enhanced behaviour of the hens on the runs corresponded to the reduced fearfulness recorded in the birds in this study. This agrees with the observation of Biasato et al. [72], who recorded a nexus between fearfulness and behaviour repertoire in broiler chickens.

In line with the observation of [68], there was no significant difference in the incidence of pecking behaviour in all the housing types. However, this is in contrast to previous studies, implying that housing type had a significant effect on feather-pecking behaviour. It has been observed that hens are highly motivated to use perches and nest boxes [[73], [74], [75]] and that they suffer from reduced welfare when they are absent. In this study, the hens with access to pasture recorded a higher frequency of perching and nesting. This may be due to the fact that they are motivated to perform this behaviour as a result of having access to runs.

Overall, the current study showed that birds kept in deep litter spent substantially more time feeding than birds with access to pasture. A possible explanation for the decreased feeding behaviour is that the complexity of the environment in the run made the hens spend a comparably shorter time feeding. On the other hand, the hens in the deep litter did not have access to run. This may have resulted in higher feeding frequency. The behavioural observations made in this study were similar in legumes and grass pastures.

The respiratory rate measures the health status and stress in broiler chickens [76]. The normal respiration rates of adult birds will range between 20 and 59 breaths per minute [77]. Although it is known that panting assists in losing heat [78], earlier reports indicated that increased panting ultimately reduced blood bicarbonate availability for eggshell mineralization, negatively affecting egg production in hens [79]. The respiratory rates of the hens in this study were within the normal range of respiration rates of 20–59 breaths per minute [77]. Our observation indicates that rearing laying hens on grass- or legume-based pastures did not alter the respiratory rates of the hens throughout the laying phases.

The housing systems of chickens can influence their physiological status [80]. Variations in the levels of stress have been associated with the housing type [60,81]. Rectal temperature has been proposed as a measure of the degree of heat stress in broiler chickens and is one of the most significant physiological reactions that reflects the thermoregulation of animal bodies [82]. According to Edgar et al. [83], it depicts the equilibrium between heat gain and loss in broiler chicks. The similarity in the rectal temperature of the hens at the early and late phases of laying in the present study suggests that the housing type did not have adverse effects on the birds’ core body temperature. It is interesting to note that the rectal temperature of the chickens on the pasture was higher than that of the deep litter system at the peak of lay (15–28 weeks in-lay) in this study. This observation corroborates the report of Olanrewaju et al. [84], who indicated that housing type influenced the body temperature of chickens. This is also in consonance with the report of Onagbesan et al., [12]. The slight increase in body temperature could be due to the exposure of the birds to the weather elements on the pastures. There is a scarcity of scientific data on the rectal temperature of hens raised on grass or legume-based pastures.

Triiodothyronine is a crucial thyroid hormone for controlling energy metabolism and preserving thermogenetic homeostasis [85,86]. Earlier findings have indicated that triiodothyronine reduced under harsh environmental conditions [[87], [88], [89], [90]]. An increase in T_3_ is a good indicator of high metabolic rates [103]. In the present trial, it is interesting to note the similarity in the T_3_ concentration in the plasma of the hens kept in the deep litter and free range in some weeks of the trial indicates a similar metabolic rate in the birds. This is partially in agreement with our earlier study, where dietary incorporation of *Stylosanthes hamata* did not influence the metabolic hormones during some weeks of growth in broiler chickens [91]. However, the significant difference in the T_3_ levels in some weeks of the trial did not follow a consistent pattern. At some weeks, higher levels were recorded in the plasma of the hens in the deep litter; at some other points, the hens on the pasture, suggesting varying metabolic rates with time. This may suggest that access to different pasture types could not upregulate the thyroid of the birds in a consistent manner. In agreement with the findings of Davis et al. [92], the levels of circulating T_3_ in the current study varied with the age and egg production cycle of the hens.

Changes in the gut microbiota may have a broad effect on the productivity, feed efficiency, and health of chickens [93,94]. It has been shown that exposing chickens to graze on pastures could have a positive effect on the intestinal microbiota [94,95]. The lower intestinal bacteria count of the hens on grass pasture suggests that the grass (Cynodon dactylon) was beneficial in regulating the bacterial load of the birds. Eggshell bacterial contamination is influenced by a number of variables, including the population of bacteria in the air of the poultry house [96] and the nutrition of the birds [97]. In addition to increasing the proportion of eggs contaminated by excreta, diets that add moisture to bird excrement also increase the microbiological contamination of supposedly clean eggs [97]. In the present trial, bacterial load was higher on the eggs laid in the different pastures than those of the deep litter. Similar to our findings, Jones and Anderson and Sharma et al. [98,99], observed that the microbial load on eggshells varied according to the housing conditions. Several pathogenic bacteria, including *Campylobacter* spp.*, Escherichia Coli, Staphylococci* spp.*,* or *Salmonella* spp. may be present in the cloaca, and the eggshell may be contaminated by these bacteria when the egg passes through the cloaca [100]. The cloacal microflora of the hens on the pastures might have been influenced by the forages or soil ingested by the birds. Earlier studies on alternate housing conditions revealed that hens reared on the runs had greater eggshell bacterial load than those kept in cages or enclosed spaces [35,99,101]. It may also be due to the fact that some eggs were laid on the pasture, where they had direct contact with the soil. One would expect that there would be a higher bacterial load in the gastrointestinal tract of the hens raised on the pasture. Interestingly, there was a higher count of bacteria in the crop of chicken kept in the deep litter than in legume and grass pastures. This is at variance with the findings of Onagbesan et al. [102], who reported that bacterial counts in the crop of hens raised in the deep litter were lower than those in the free range. The discrepancy may be ascribed to the litter materials of the hens. In the deep litter system, the bedding material, mixed with faeces, creates a warm, moist environment ideal for bacterial growth. Additionally, the improved behaviours of the hens on the pasture could help get rid of the bacteria and parasites on the birds, thereby reducing their bacterial load.

While this study has provided some valuable insights into the impact of access to legume- or grass-based pastures on the behaviour, physiological responses, and bacterial load of laying hens, certain limitations are acknowledged, including changes in the environmental conditions during the study, as the study was not conducted in a controlled environment. Future research is recommended in a controlled environment. Moreover, the study did not assess the specific types or strains of bacteria present; thus, further research is necessary to understand the implications of access to pastures on the presence and transmission of various pathogens.

## 6 Conclusion

To conclude, access to pasture affected the behaviours of hens and the duration of tonic immobility was shorter in the hens on the pasture, suggesting that access to pasture improved the welfare of the hens. However, the bacterial load on the eggs of the hens reared on the different pasture types was higher than that of the deep litter housing type. Moreover, the access to legume- or grass-based pastures encouraged some natural behaviour repertoires in laying hens. Further studies are needed on the inconsistency of the metabolic hormones of hens raised on legume- or grass-based pastures.

## Ethics statement

The study was approved by the Animal Care and Use Committee of the College of Animal Science and Livestock Production, Federal University of Agriculture, Abeokuta, Nigeria with the reference FUNAAB/COLANIM/ACUC/2022/07. The study complied with all the regulations of the committee.

## CRediT authorship contribution statement

**O.E. Oke:** Writing – review & editing, Writing – original draft, Visualization, Validation, Supervision, Software, Resources, Project administration, Methodology, Investigation, Funding acquisition. **O.M. Onagbesan:** Writing – review & editing, Visualization, Validation, Software, Resources, Project administration, Methodology, Funding acquisition, Formal analysis, Conceptualization.

## Declaration of competing interest

The authors declare that they have no known competing financial interests or personal relationships that could have appeared to influence the work reported in this paper.
